# Piecing together the puzzle: Improving event content coverage for real-time sub-event detection using adaptive microblog crawling

**DOI:** 10.1371/journal.pone.0187401

**Published:** 2017-11-06

**Authors:** Laurissa Tokarchuk, Xinyue Wang, Stefan Poslad

**Affiliations:** 1 Cognitive Science Research Group, School of Electronic Engineering and Computer Science, Queen Mary, University of London, London, United Kingdom; 2 Centre for Intelligent Sensing, School of Electronic Engineering and Computer Science, Queen Mary, University of London, London, United Kingdom; Tampere University of Technology, FINLAND

## Abstract

In an age when people are predisposed to report real-world events through their social media accounts, many researchers value the benefits of mining user generated content from social media. Compared with the traditional news media, social media services, such as Twitter, can provide more complete and timely information about the real-world events. However events are often like a puzzle and in order to solve the puzzle/understand the event, we must identify all the sub-events or pieces. Existing Twitter event monitoring systems for sub-event detection and summarization currently typically analyse events based on partial data as conventional data collection methodologies are unable to collect comprehensive event data. This results in existing systems often being unable to report sub-events in real-time and often in completely missing sub-events or pieces in the broader event puzzle. This paper proposes a Sub-event detection by real-TIme Microblog monitoring (STRIM) framework that leverages the temporal feature of an expanded set of news-worthy event content. In order to more comprehensively and accurately identify sub-events this framework first proposes the use of adaptive microblog crawling. Our adaptive microblog crawler is capable of increasing the coverage of events while minimizing the amount of non-relevant content. We then propose a stream division methodology that can be accomplished in real time so that the temporal features of the expanded event streams can be analysed by a burst detection algorithm. In the final steps of the framework, the content features are extracted from each divided stream and recombined to provide a final summarization of the sub-events. The proposed framework is evaluated against traditional event detection using event recall and event precision metrics. Results show that improving the quality and coverage of event contents contribute to better event detection by identifying additional valid sub-events. The novel combination of our proposed adaptive crawler and our stream division/recombination technique provides significant gains in event recall (44.44%) and event precision (9.57%). The addition of these sub-events or pieces, allows us to get closer to solving the event puzzle.

## Introduction

Since the emergence of Web 2.0, the way people engage with news events has been fundamentally redefined. Instead of passively consuming online news, the general public is actively involved in reporting and commenting on different kinds of news events. They post observations and express their opinions through social media services such as Twitter. Their comments and sentiments on a range of events such as sports competitions[1, 2], festivals [3, 4], political elections [5–7], emergencies [8] and global disasters [9–11] have been analysed by researchers to determine the timeliness and effectiveness of Twitter on reporting these newsworthy events.

**Fig 1:**
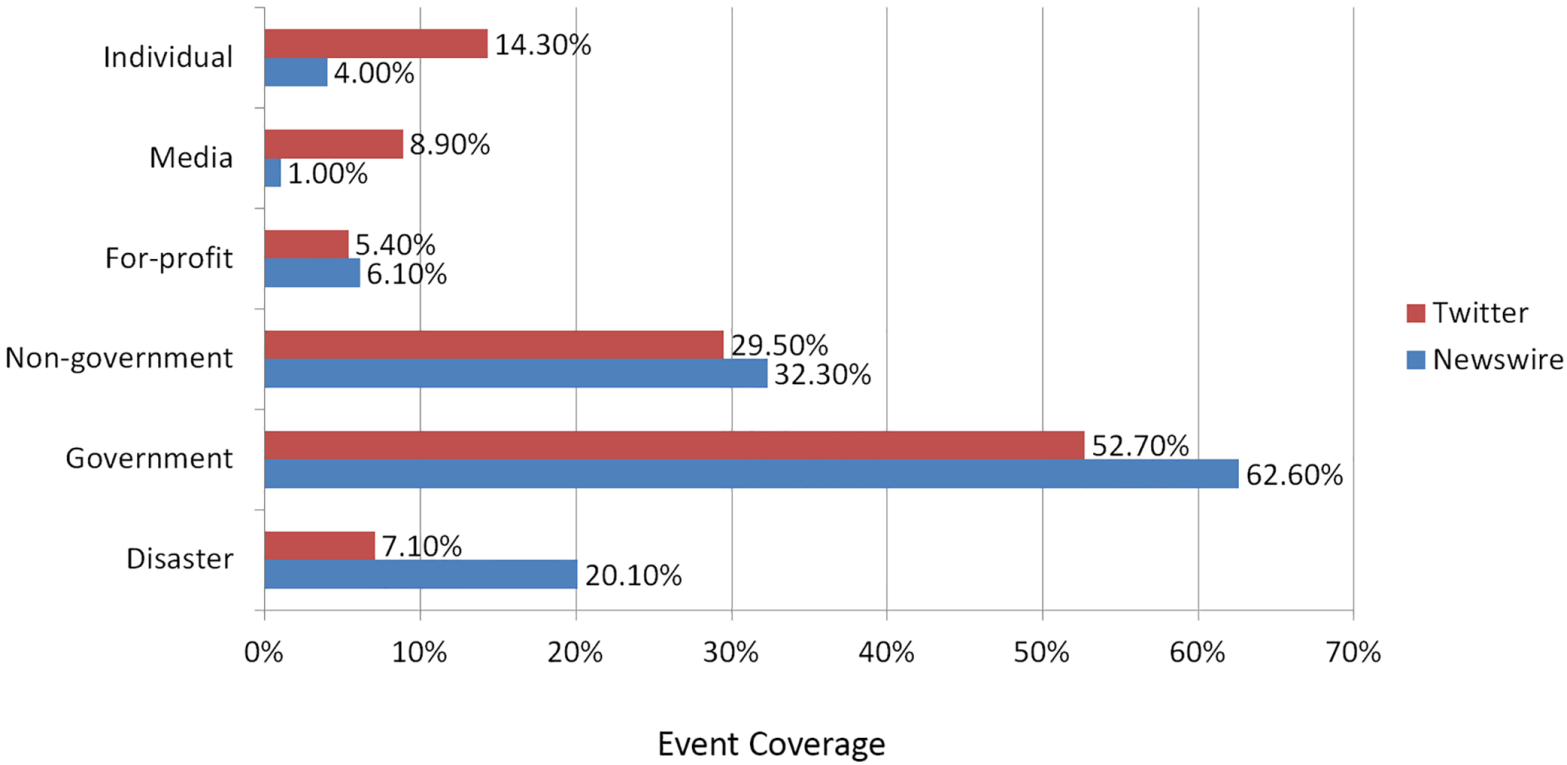
Distribution of events coverage in main stream newswire and Twitter (based on [20]).

In the last decade, Twitter has been exploited to report the first story of the real-world events. Empirical studies have shown that Twitter is becoming a prominent communication tool for breaking news dissemination, which reveals both the broadcast events [12, 13] and local affairs [14, 15]. Since Twitter is an open platform that reports anything people feel inclined to share, it is full of posts about their daily activities, i.e. where they went, what they saw [16], advertisements for products and services [17] or rumours about celebrities or events [18], the initial attempts aim at filtering out the noise and discovering the first informative event tweets from the massive and noisy Twittersphere. Recent research has shown that Twitter leads the traditional online newswires not only by reporting sports and disaster events more efficiently [19], but also by providing broader coverage of event information [14] along with additional viewpoints [20, 21]. For instance, [20] compare the main stream newswire against Twitter in regard to the discussions on climate change events, as shown in Fig 1, where the y-axis represents the categories of events. It can be observed that while the newswires act as the microphone for the government, Twitter provides significantly much information about media or individuals. Rather than waiting the reports provides by the lengthy and labour intensive production pipeline followed by the traditional newswires (i.e. acquiring, writing, reporting and producing [22]), more and more people favour of scrolling through the Twitter timeline and tracking live Twitter streams for breaking news [23]. However, while Twitter contains a copious amount of information, getting at the information is not trivial. Twitter is well known for containing repeated information (inherent in the retweet functionality), rumour [24] and noise. Automatically detecting Twitter events enables for the possibility of collecting information about both the events and the underlying stories (or sub-events) in order to get a fuller picture of an event. Current techniques in event detection do not address the problem of rumours or noise, but rather help to reduce the volume of information.

To monitor the finer granularities, i.e. sub-events, of an event, the common practice is to analyse a filtered Twitter stream using event detection approaches that are developed for conventional Topic Detection and Tracking tasks [25]. A large proportion of these solutions detect different phases of disaster events for offering situational awareness to different stakeholders in the process [26], such general public and government authorities. Other research work investigates the sub-events of planned events, such as sport competitions [27, 28] and festival activities [3]. However, these solutions suffer from three kinds of limitations. First, most of the sub-event detection algorithms are infeasible to run in real-time. To better detect and understand sub-events, researchers upgrade the existing solutions by running them with multiple iterations. This is at the cost of extra complexity and additional resources. Second, most of the event detection algorithms are designed and evaluated for a particular type of event. They either assume that prior knowledge exists or require specific features to exist in the input data. Third, almost all the detection algorithms analyse events with a static set of tweets that is specified by one or multiple keywords. However, in the real-world scenario, events evolve and adapt while they develop, and new stories relating to the event of interests constantly emerge. For example, during the protests and public movements of the Ukraine crisis (i.e. a political movement between Ukraine, European Union and Russia), new tweets and reports about the crashing plane, i.e. the Malaysia Airlines Flight 17 (i.e. a plane belongs to civil aviation company that are wrongly shot down by military), were posted immediately after the casualty.

As a result of the drawbacks of the existing solutions, a research gap exists in supporting the real-time sub-event detection in order to provide timely sub-event detection and comprehensive descriptive information about the underlying stories. Consequently, the focus of this research is on proposing a real-time sub-event detection solution that identifies and summarises the sub-events by analysing a comprehensive set of event content. Namely, rather than extracting newsworthy events from massive Twitter stream as done in the existing work on First Story Detection [29–31], the major contribution of this paper is the Sub-evenTs detection by Real-time mIcroblog Monitoring (STRIM) framework that detects and summarizes the sub-events from a live expanded event stream (i.e. a Twitter stream about a particular news event which is specified with user-input keywords, but which adaptively adjusts in real-time to ensure expanded event coverage). To accomplish this, the proposed solution deals with two common challenges in Twitter event detection, namely 1) the processing of high-arrival-rate tweets and 2) generalisation of the solution as to require no a priori knowledge about the event. It accomplishes this by only requiring keywords that do not require a priori event knowledge. It maintains event integrity and coverage by automatically expanding the initial keywords while still restricting the arrival stream to a manageable rate. This paper also makes novel contributions regarding to the sub-event detection solution. First of all, to our best knowledge, this is the first work that uses expanded streams in sub-event detection and which evaluates the impact of the extra event content in the real-time sub-event detection task. Previously, event detection solutions made their conclusions based only on tweets retrieved by a static set of pre-defined keywords. Additionally, in order to take advantage of the extra event content identified by the adaptive microblog crawling in our previous work [32], this paper deploys the existing event detection algorithm in a parallel manner.

The rest of this paper is organized as follows: Section 2 introduces the related work from the perspectives of tweets retrieval, event detection and summarization. Then, the overview of the proposed event monitoring framework is outlined in section 3. Section 4 presents the techniques and methods employed. Section 5 provides the experimental details and results. Finally, Section 6 suggests some future direction based on the results in this paper.

## Related work

Exploiting Twitter for news event monitoring has been a research hotspot in the recent decade and intensive research efforts have been made. Before exploring the relevant literature in this domain it is important to provide definitions for the key terms used in this area and subsequently in in this paper. The formal definition of event and sub-event are given as follows:

Definition 1: an **event** under social media environment is something that happens in the real-world at a certain time period and receives constant discussion by social media users during that time period.

Definition 2: an **event stream** is a set of temporal coherent text pieces that are overlapped in vocabulary. The overlapped vocabulary concerns a common event in social media. An event stream can be represented as a sequence of tuples that includes a timestamp and a set of features, or terms.

Definition 3: a **sub-event** describes the episode of an event by the underlying story or sub-sequent story. The content of sub-events belonging to the same event made up the event stream. As a result, each sub-event can also be represented by tuples which are strongly correlated with each other by the features and coherent by the timestamps.

This section reviews the existing practices on Twitter sub-event detection. Specifically, it will examine the related work on event tweets retrieval and Twitter sub-event detection.

### Event tweets retrieval

Twitter stream is very dynamic and noisy. Apart from the event information about breaking news, the stream also consists of people’s daily chat, advertisements and rumours. Since the huge amount of noise co-exist with event information about sports competitions, festivals, political elections and global disasters, it is necessary to minimise the amount of noise prior to the event detection. This can be achieved by retrieving tweets by matching it with specific condition, e.g. event terms and keywords. Rather than used the random sampled data that available from Twitter's Streaming API as done in early research [33–35], our research retrieves the event-relevant tweets by using a focused crawler, i.e. matching tweets with pre-defined properties and features. Among the research work analysing the Twitter stream, common tactics to get specific information relating to an event has been achieved by collecting based on tweet properties or Twitter symbols, such as keywords and #hashtags [1, 6, 27], @mentions [36], and URL links [37].

In addition to the Twitter symbols, attempts to retrieve event-relevant tweets by exploiting data from other sources have also been made. For example, Becker, Iter, Naaman and Gravano examine the use of precision and recall-oriented strategies to automatically identify event features for updating previous queries to retrieve additional event content from diverse social media sites [3]. Their approach relies on event announcements from Eventbrite to formulating a more precise query in order to achieve higher retrieval recall. Some researchers use the same idea but rely on the semantic web. They associate tweets to a given event using query expansion techniques with related terms that are defined on Semantic Web.

### Sub-event detection from Twitter Stream

For sub-event detection, some researchers apply supervised learning techniques used in Natural Language Processing, such as Conditional Random Field and Named Entities Recognition [38]. Researchers have also shown that the detection can be achieved through ontologies from semantic web [4]. Approaches that are based on supervised learning of the temporal-spatial features of tweet have also been proposed [39, 40]. In all of these tasks however, researchers have made an assumption that the event is known in advance and thus can be learned from existing resources or materials. In this research, the objective is to generalize the solution for different kind of event without any prior knowledge.

The most common practice used for Twitter sub-event detection is based on the burst detection approaches. The fundamental idea in this approach is that an emerging news event quickly attracts people's attention and this is evidenced by a sudden increase of posts and forwards about it, and therefore can be associated with the burst of posting or words frequency [41–43]. In the early research, for example, Earle at el. compare the short-term-average of tweets volume to the long-term-average for identify possible earthquakes [41], as well as the sport events [42] and political affairs [43]. The Twitinfo system detects events using a similar technique based on a more theoretical model. Instead of manually defined how to calculate the average, their work borrows the idea of exponential weighted moving average from TCP's congestion control mechanism for smoothing the frequency count to enable a better event detection [11]. Later research exploits finer granular features, such as the frequency of event terms, for better sub-event detection results. For instance, Aiello et al. and Li, Sun and Datta use complex hierarchical clustering methods [6, 34] to merge the fragment term features into meaningful sub-event clusters. In [35], researchers propose modelling the event stream as a mixture of multiple topic models. While this solution achieves good results it requires a priori knowledge regarding the number of sub-event to be detected and thus cannot be applied to the real-time sub-event detection task. The other solutions discussed above also rely on fairly complex techniques that dramatically increase the processing cost for the detection and thus cannot be directly applied to the scenario of this research. In addition, most of the sub-event detection algorithms are tailored to deal with particular type of events, i.e. the analysis is based on multiple instances of the same event. For example, [27] only evaluate their system on game of the FIFA World Cup and [8] test their solution on the Red River floods.

### Sub-events detection by Real-time microblog monitoring (STRIM) framework

The STRIM framework detects and summarises sub-events surrounding a specific specified topic, such as an earthquake or sports event. In order to accomplish this, we propose an integrated solution for sub-event content retrieval, detection and summarization by extending existing event detection research. Whilst existing research focuses on the depth of detection, i.e., on more accurate detection results with sophisticated but inefficient algorithms, the focus of this research is to achieve higher accuracy on sub-event detection by increasing the coverage of the event content. By collecting, detecting and then extracting the most descriptive event-relevant tweets, the framework automatically formulates descriptions of the sub-events.

**Fig 2:**
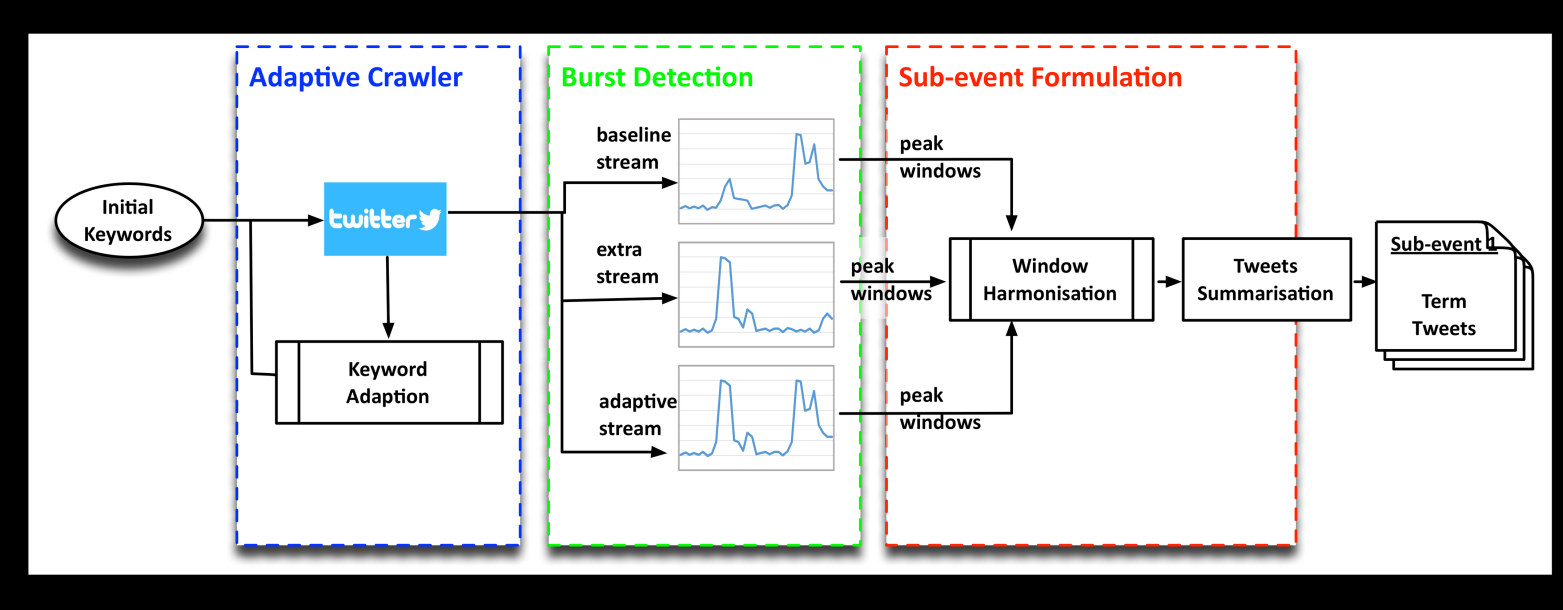
STRIM framework for TEM.

As shown in Fig 2, the proposed event monitoring framework contains three main components. The **Adaptive crawler** component collects a comprehensive set of event tweets by adaptively improving the Twitter search terms in real-time. It produces a stream of event tweets that are analysed by the Parallel **Bursts detection** component. The bursts detection component identifies the potential sub-events, where each potential sub-event is represented by a timespan and a collection of tweets whose timestamp falls within the timespan. After the detection of peak window, all the potential sub-events are post processed by the **Sub-event formulation component** to finalise the description of the detected sub-events. This framework is initiated by a set of user-specified keywords that describe the target event. The stream collected from solely from these user-specified keywords forms the baseline stream, the stream collected using the adaptive crawler forms the adaptive stream and finally the unique tweets collected only from the adaptive crawler (adaptive stream–baseline stream) form the extra stream. The final output is a list of sub-events which are specified by a timespan, a group of descriptive terms and a summary tweet.

### Adaptive crawler

A Twitter crawler is a program that collects tweets or users' information through the Twitter API by matching a set of search criteria. Although Twitter provides multiple parameters to track with, keyword tracking is the most commonly used approach in real-world event detection since the context and semantic information of a tweet affect its relevance to an event [44]. Existing event monitoring solutions rely on data collected from conventional keyword Twitter crawler. They define and use a constant keyword set to crawl a collection of tweets about a particular event. The keywords used for collection remain unchanged for the entire collection period. In this paper, the stream collected by this kind of baseline crawler is referred as baseline stream.

Different from the existing solutions, this paper uses our adaptive crawling model [45] to improve the comprehensiveness and quality of event content coverage. In the adaptive crawling model, the tweets retrieval process is triggered by the same set of predefined keywords as the baseline crawler. This model enables the keyword adaptation feature so it identifies event topics that arise in the midst of events and timely adjusts the search query to include these newly emerged key terms. Our previous work has explored several types of keyword adaption algorithms and concluded that our Content Similarity Keyword Adaptation Algorithm (CS-KwAA) achieves the best performance in terms of achieving significant coverage with minimal noise. This algorithm exploits the content similarity between different hashtags [46]. The addition of these search terms has been shown to increase both the amount of event relevant information collected and the number and size of tweet bursts (an important characteristic used in event detection) [45, 46]. Specifically, at the end of some user defined time frame, the algorithm selects high frequency hashtags that co-occur with the initial pre-defined keywords. After that, for each high frequency hashtag, the algorithm retrieves a collection of the most recent tweets. These collections of tweets for each hashtag are converted into TF-IDF vectors, i.e. hashtag-based TF-IDF vector. By calculating the similarity between hashtag-based TF-IDF vector of high-frequency hashtags and hashtag-based TF-IDF vectors of the initial keywords, hashtags with similarity higher than a defined threshold (Thres) are considered to be new keywords. Finally, a query that encodes all the new keywords and the initial keywords is sent to the Twitter API. This process repeats after every time frame. As a result, the output of this adaptive crawling model is the adaptive stream about event of interest and a set of extra keywords identified by the KwAA.

The adaptive crawling model used in this paper is written in Java and uses the open third-party application-programming interface Twitter4J to connect to Twitter’s Streaming API to collect tweets based on the defined keywords. The software stores the collected tweets in an SQL database. It then processes the tweets to identify potential keyword candidates, queries Twitter’s Search API to gather historical usage about them and then uses Apache Lucene’s Twitter Analyser and Apache Mahout to process and generate the TF-IDF vectors.

### Parallel burst detection

Detecting the outlier or abnormal peak over Twitter traffic is commonly used in the sub-event detection problem. This kind of approach is also known as burst detection. One common observation of the Twitter traffic during the news events reveals that the burst of features is positively correlated with the occurrence of a real-world trending event. Therefore, these burst detection algorithms are designed to identify the sub-events by capturing the sudden bursts of tweets features.

In this component, an existing burst detection algorithm used by Twitinfo system [11] is parallelised to run over multi-threaded event streams to improve the detection of potential sub-events. The original algorithm is inspired by the conventional statistical model based outlier detection algorithms but improves the detection performance by using a smoothing technique. Specifically, the algorithm exploits the exponentially weighted moving average (EWMA) in TCP congestion control [47] to identify the relatively maxima, i.e. burst, by considering the recent history. Although the EWMA is designed to find the outlier of package arriving rate in a communication channel, it can be used in the event detection task to distinguish local extreme values in the tweet arrival rate. In general, this algorithm identifies a peak window or event when it encounters a significant increase in tweet volume. The end of the peak window or event is identified as when the tweet volume returns to the same level as before the burst started. Consequently, a **peak window** is a pair of timestamps, where the first timestamp defines the moment when the detected event starts and the other one defines the moment when that ends.

To maximise the utilisation of the extra event content identified by the adaptive crawler, our experiments show that the burst detection algorithm should be applied not only to the adaptive stream, but also to the extra-only stream. The reason behind this is to minimise the noise from the original stream in the sub-event detection task. While significant amounts of information are collected about these sub-events by the adaptive crawler, in some cases the total volumes are not significant enough to for the event to be detected if included with the original Twitter stream. As a result, this STRIM framework applies three instances of the Twitinfo event detection algorithm in parallel over the three decomposed stream, i.e. the baseline stream, adaptive stream and the extra stream, as detailed in Algorithm 1. Here, the baseline stream is made of event content identified by the baseline crawler with the same initial keyword as the adaptive crawler. The extra stream is composed by the tweets that can be identified by the adaptive crawler but not the baseline crawler, i.e. the stream obtained based on the Eq 1. For any of the decomposed streams, a list of peak windows is generated and sent to the next step.

extrastream=adaptivestream-(adaptivestream∩baselinestream) (1)

**Algorithm 1** Multi-threads Twitinfo

**for** ∀ tweet *t* of the incoming tweets

 **if** *t* contains any *h*_*k*_ *∈ H*_*seed*_

  add *t* to baseline stream (*BL)*

 **else**

  add *t* to adaptive stream (*AD)*

  **if** any *h*_*k*_ *∈ H*_*seed*_ not exists in *t*

   add *t* to extra stream (*EX)*

  **end if**;

 **end if**;

**end for**;

**for** ∀**t_stream_** ∈ {BL, AD, EX}

 WL_BL/AD/EX_ = {W_1_, …,W_n_} <- apply Twitinfo algorithm on t_stream_

**end for**;

### Sub-event formulation

The lists of peak windows (WL_BL_, WL_AD_ and WL_EX_), i.e. output from the parallel burst detection component, don’t provide the context information about the sub-events. They only demonstrate the timestamp when there is abnormal burst on tweets volume. As a result, this research extracts the textual information from the AD, BL and EX for describing all the detected peak windows. The number of tweets retrieved for any peak window is still very large and thus representative tweets from this window are chosen. This process consists of two sub-steps are involved in this sub-event formulation process:

#### Window harmonisation

Each peak window *W* that is detected from one of the Twitter stream among BL, AD and EX can be described by a tuple *W = {t*_*s*_, *t*_*e*_, *T}*, where t_s_ and t_e_ specify the start time and the end time of the peak window respectively. Based upon the set of tweets *T* that is retrieved during the window, the STRIM identifies the most frequent unigram (*FreqUni(T)*), measured by their TF-IDF value. The assumption here is that the summary term of one window should be very different from other windows. Therefore, the TF value is calculated by all the tweets in window *W*, and the IDF is based on the tweets in all the previous identified peak windows. Following the same strategy as in the Twitinfo system, terms with highest TF-IDF value from each peak window are selected to represent the corresponding potential sub-event. We follow the same strategy used in Twitinfo system, set the number of summary terms (*ST*) to *N = 5*. Since the burst detection algorithm is applied in parallel to all three decomposed streams, as shown in Fig 2, three peak window lists are generated after the bursts detection procedure. As a result, when running the burst detection algorithm over multiple streams, there is probability that peak windows from different event streams represent the same sub-event. In the proposed framework, a window combination step is employed to reduce the amount of duplicated peak windows. In this sub-step, two peak windows that are detected from different streams are considered as duplicated if the peak windows overlap in time and contain more than half common summary terms. Once the two windows are recognized as duplicated, they are combined together and considered as single peak window. We use the same properties, i.e. timespan and summary terms to describe the combined window. The new timespan is calculated as the union of the individual timespan of all the duplicated windows, while the summary terms are recalculated based on the same strategy for the summary selection process which is used in the Twitinfo system.

#### Summary tweets

This research takes advantage of tweets in the peak windows for a structured summarization. We use the TF-IDF value of a tweet for selecting summary tweets as it is a common practice [11, 28]. A recent study also concludes that the simple frequency based summarisers produce the most promising performance when selecting tweets for event summarization [48].

In this approach, the score of a tweet is the average TF-IDF value of all the terms that appear. Specifically, this final score, i.e. *normalized TF-IDF score ($\bar{TF-IDF}$)*, is calculated by Eq 2.

$$\bar{TF-IDF}=\frac{1}{n}\sum_{i\;=\;1}^{n}{tfidf}_{i}$$ (2)

Before the calculation, all the tweets are pre-processed with stop word and punctuation removal, stemming and Twitter symbols (@ user mention and shorten URL) filtering, where the remaining terms are referred as *informative terms*. However, the drawback of using the normalized TF-IDF score is that this strategy favours selection of short tweets that only contains few terms with very high TF-IDF value. Normally, the number of the distinct informative terms in the summary tweet is often less than 2, being primarily made up of terms with high TF-IDF values. Since these short length tweets typically don't provide any extra information than the summary terms, this research preferentially selects tweets with more different terms. Specifically, the summary tweets is expected to have the highest normalized TF-IDF score among all the tweets belonging to that peak window and have at least two terms. A longer tweet with the same normalized TF-IDF score as a shorter tweet will always be preferred.

**Algorithm 2** Summarization with Multi-streams

**input** Peak window lists WL_BL_, WL_AD_, WL_EX_, Top tf-idf unigram N

**output** sub-event lists WL_event_

//1.Window Harmonisation

**for** ∀ W_x_ = {t_sx_, t_ex_, T_x_}∈ {WL_BL_, WL_AD_, WL_EX_}

 ST_x_ = Top N in FreqUni(W_x_,(WL|∃ W_x_∈ WL),x)

 **for** ∀W_y_ = {t_sy_, t_ey_, T_y_} ∈ {WL_BL_, WL_AD_, WL_EX_} and

    W_x_, W_y_ not belong to the same peak window list

  ST_y_ = Top N in FreqUni(W_y_,(WL|∃ W_y_∈ WL),y)

  **if** t_ex_ **>** t_sy_ or t_ey_ **>** t_sx_ and len(ST_x_ ∩ ST_y_) > N/2

   W_x_ and W_y_ are duplicated

   WL_temp_[0] = {min(t_sx_,t_sy_), max(t_ex_,t_ey_),T_x_+T_y_}

  **else**

   WL_temp_[0] = W_x_, W_temp_[1] = W_y_

  **end if**;

  add W_temp_ to WL_event_

 **end for**;

**end for**;

//2.Summary Tweets

**for** ∀ W_i_ ∈ WL_event_

 **for** ∀ t_i_ ∈ T_event_

  **if** score < $\bar{TF-IDF}(t_{i})$

    score = $\bar{TF-IDF}(t_{i})$

   summary(*W*_*i*_) = *t*_*i*_

  **end if**;

 **end for**;

**end for**;

**function** FreqUni(W, WL,idx)

 **for** ∀*term*_*i*_*∈ T*

  tfidf(W,term_*i*_) = tf(T)∙idf(W1, …, Wn) where n ≤ idx

 **end for**;

**return** sort(tfidf(W,term_1_),…, tfidf(W,term_*i*_))

## Experiments

STRIM is unique in its use of expanded, adaptively collected event information and its streamed parallelized burst detection. The objective of the experiments in this section is to demonstrate that STRIM achieves a greater breadth of event detection and, as a result of its more detailed data, allows for better event summarization. This section first introduces the datasets, algorithm parameters and evaluation methodology before presenting the results and discussing the effectiveness of the proposed sub-event detection solution.

### Experiment setup

#### Datasets

This paper focuses on the use of an event stream related to a particular type of event and not the whole Twitter stream. This event stream is collected with a small set of user-selected keywords. To explore the detection benefits under different event scenarios, this research selects two events for the investigation: the *2013 Glastonbury Music Festival* (Glastonbury Festival) and the *2014 Sochi Olympic Games* (Sochi Olympic). The Glastonbury festival event consist of densely back multiple sub-events because multiple performances are carried out simultaneously. On the other hand, the schedule of the Sochi Olympic event is much more sequential in nature and even irregular because the time duration of each competition varies. A detailed overview of the evaluated datasets is shown in Table 1. Both the baseline crawler and the adaptive crawler are triggered by the same set of initial keywords. Since the adaptive crawler identifies extra keywords during the developing of the event, the number of keywords of adaptive crawler is significantly higher than that of baseline crawler, by 117 and 244 for Glastonbury Festival and Sochi Olympic respectively. Data was collected during the relevant periods by connecting to Twitter using the public interface provided by Twitter-4J. This interface accesses the freely available 1% stream. All data collected complied with Twitter’s terms of service.

**Table 1:** Overview of the Glastonbury Festival and Sochi Olympic datasets.

	Glastonbury Festival		Sochi Olympic	
	Baseline	Adaptive	Baseline	Adaptive
Init. Keywords	Glastonbury		Sochi, #olympic2014, #sochi2014	
Period	2013-06-29, 11:00 to 2013-06-30, 00:00		2014-02-22, 05:15 to 2014-02-22, 19:15	
Tweets No.	171254	232811	213986	281692
Keyword No.	1	118	3	247

#### Sub-event ground truth

Table 1 demonstrates that the tweets that are associated with the pre-defined news event can be very large. Consequently, the total number of sub-events that are hidden in the datasets can be numerous. Pre-identification of all the sub-events from an event datasets is often infeasible due to the number and range of such events. Considering that our research is interested in the identification of newsworthy sub-events, we define the ground truth based on the reports in mainstream online news media. Specifically, a detected sub-event is considered as newsworthy if the entities in the summary tweets shows together with the retrieved keywords in the headline or content of a news report or Wikipedia page. Furthermore, although we use this metric to judge STRIM, it is important to note that this is done on a historical basis many months later and that if applied to a live stream, STRIM would detect many events, some of which will eventually be newsworthy and some of which won’t. In truth, it is the events which are possibly not newsworthy that will be the most interesting, however in testing and evaluating STRIM there is no way to measure this. The list of the newsworthy sub-events that are existing and detectable from the all datasets is summarized in Table 2.

**Table 2:** Sub-event ground truth for Glastonbury Festival and Sochi Olympic.

Sub-event indexes	Sub-event lists	
	Glastonbury Festival	Sochi Olympic
1	Ben Howard	Discussion about the championship of Kim Yuna
2	Laura Mvula	Ice hockey Canada vs. USA
3	Tibetan Monk Throat Singing	Vic Wild for men’s snow board
4	Elvis Costello	Photo of the day: three Olympic champions
5	Noah and the Whale	Mao Asada memory and feature report by Asahi Shimbun
6	Primal Scream	Speed Skating champion by Netherland
7	Maverick Sabre	Plushenko Back Surgery
8	Glastonbury founder supports badger cull	Anton Shinpulin led the Russian team to win the biathlon relay
9	Two Door Cinema Club	Ice Hockey USA vs Finland
10	Example	
11	The Rolling Stones	

#### Parameter setting

There exist multiple parameters that need to be tuned in the STRIM framework. For the threshold of adaptive crawler, we follow the same strategy used in the previous research [46], that is Thres = 0.8. The other parameters need to be tuned are those used for the burst detection component: the detection latency p, the sample interval t, the smooth factor alpha and the inequality threshold tau. Although it is possible to adopt the original setting directly (p = 5, one-minute sample interval, alpha = 0.125, tau = 2), a universal parameter setting for all the events is not appropriate due to the nature of different events. This is because the variation of tweet volume can be event specific. As a result, this research determines these parameters using a heuristic approach by considering the characteristics of each event with statistical and empirical observations. This research chooses p to a value that covers a constant period of time, i.e. t_period_ = 30 minutes, for all the evaluation. By employing a fixed time period, the value of parameter *p* can be calculated with formula p = *t*_*period*_*/t*_*sample*_. An empirical experiment on different sample interval proves that the intensive variation of volume brought in by a shorter time interval can be balanced by providing the algorithm with alpha value less than 0.5. In other words, when the sample interval is decreasing, alpha need to be reduced in order to obtain a similar detection result. The sample interval is determined on an event basis: 5 minutes for Glastonbury Festival as this is the average length of a song during the performance and 10 minutes for Sochi Olympic as this is the minimum time length for the competition that day. For the setting of alpha and tau, this research explores the number of window that can be detected for each pair of them. The parameter tuning process is based on the historical frequency count on the tweets volume. A ground truth for the location and length of peak is generated based on visual analysis of the historical tweet volume plot. By comparing the detected windows against the ground truth, the parameters are set with values that enable the highest detection accuracy (all the bursts are detected even after smoothing). In fact, the values of these parameters are more dependent on the sample interval rather than the characteristic of event. The final value of each parameter is shown in Table 3.

**Table 3:** Parameter settings of Twitinfo burst detection algorithm.

	Glastonbury Festival	Sochi Olympic
Detection latency *p*	6	3
Sample interval *t*_*sample*_ (in mins)	5	10
Smooth factor *alpha*	0.6	0.75
Inequality threshold *tau*	2.5	2.75

#### Considered methods

In the Twitinfo system, the input of the event detection algorithm is the baseline stream retrieved by the baseline crawler with a set of fixed and pre-defined keywords. Thus the quality of the detection results is very dependent on the choice of initial keywords. In our work, the keywords used for event content identification is updated periodically, thus generates the adaptive stream. In addition, we propose an event monitoring framework that decomposes the streams into three parts which have specific characteristics and allow for sub-event detection at different levels of granularity:

- **BL**: the common baseline which apply the detection algorithm directly to the pre-defined keyword specified baseline stream
- **AD**: using the same detection algorithm as the BL approach but exploiting the adaptive stream that retrieved by the adaptive crawler with the same initial keywords as BL.
- **EX**: using the same detection algorithm as the BL approach but exploiting the extra stream that obtained by Eq 1.

Running sub-event detection in parallel on each individual stream reveals more sub-events than any one single stream alone. Running STRIM on the BL stream only is the equivalent to comparing our framework with TwitInfo.

#### Evaluation metrics

This research quantifies the detection accuracy by the precision, recall and F-measure, correlated to the amount of reasonable sub-events that can be detected from the datasets. We use **event precision** (P_event_) to measure the proportion of peak windows that corresponds to real-world sub-events. A sub-event of an event refers to the underlying story that happens at a particular time period and reported via online media. For example, a peak window that is summarised by a tweet “*I think I'd be quite into Glastonbury if I was some kind of predator or serial killer*'' in the Glastonbury Festival datasets is not considered a sub-event whereas *“Moves like Jagger is now something a careworker writes on a physiotherapy report #bbcglasto*.” Clearly the former tweet is an opinion tweet and doesn't corresponding to any realistic events where the latter one gives information on the band/artist currently playing. This research defines the **event recall** (R_event_) as the proportion of distinct sub-events can be detected. When applying the burst detection algorithm on the event datasets, there is chance of detecting multiple peak windows (normally consecutively) talking about the same sub-event. If two detected peak windows are related to the same sub-event, both of them will add credit to the P_event_, but only one distinct sub-event will be considered when calculating event recall. As mentioned previously, since the manually extraction of all the existing sub-event can be non-trivial task, the total number of ground truth sub-events is defined as the sum of number of realistic sub-events can be detected from all three considered methods. To synthetically consider both the precision and recall of the detection performance, we also use the F1 measure which balance both R_event_ and P_event_ by equal weighted with their harmonic mean.

### Performance evaluation

**Fig 3:**
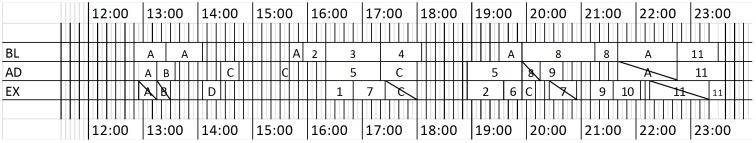
Labelled peak windows for Glastonbury Festival.

**Fig 4:**
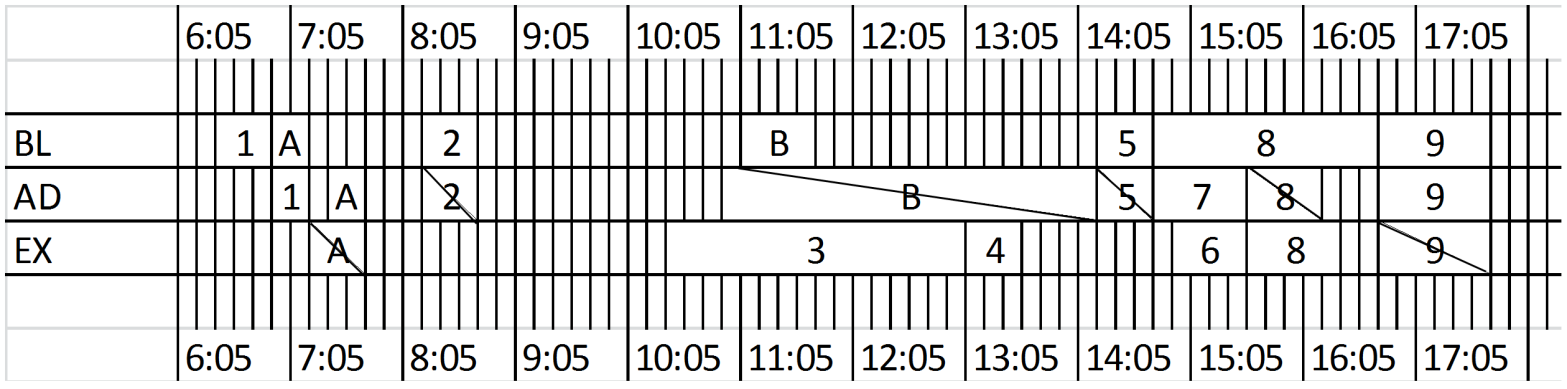
Labelled peak windows for Sochi Olympic.

The decomposed event streams, i.e. baseline, adaptive and extra stream are identified by the adaptive crawler, sampled and sent to the parallelized burst detection algorithm. For each input stream, the component identifies a list of peak windows. As demonstrated in Figs 3 and 4 for the Glastonbury Festival datasets and Sochi Olympic datasets respectively, each box (with label) in the figure represents a peak window that is identified by the burst detection algorithm. These windows and the summary tweets are then examined by at least two participates to check whether they corresponding to the real-word sub-events. Specifically, the task is to compare the summary tweets with the online resources from online social media, such as event websites, mainstream news media and Wikipedia pages. These real-world sub-events are labelled with a number in Figs 3 and 4, where the number indicates the sub-event indexes as listed in Table 2. On the other hand, the boxes that are labelled with a letter are peak windows that don’t correspond to any real-word sub-event. The windows are slashed if they are combined with other existing windows in the window harmonisation step according to the proposed event monitoring framework.

As shown in Fig 3, The BL and AD datasets of Glastonbury Festival results in the same amount of peak windows, while both are less than that of EX dataset (11 versus 14). 6 out of 11 peak windows in BL datasets correspond to the realistic sub-events, and one of them is a duplicated sub-event (two peak windows relate to 8. Glastonbury founder supports badger cull). The effectiveness of event detection algorithm over the baseline dataset can be more explicitly observed from Table 4, where the accuracy on the sub-events identification is presented. As the number of peak windows and the number of ground truth are the same, precision is higher than recall due to the effect of the duplicated sub-event. These results in the 49.59% F1 score for the BL dataset. The F1 score for the AD dataset is lower than that of BL by 9.19%. This is caused by event-irrelevant tweets. They decrease both the detection precision and recall. As shown in the AD row in Fig 3, among all the 11 peak windows of the AD dataset, 5 of them are labelled as realistic sub-event. For the remaining 6 windows, 2 of them talk about Glastonbury but can't be associated to the reports from online sources. For example, peak window 6 is summarised by a tweet “*I think I'd be quite into Glastonbury if I was some kind of predator or serial killer”* in the Glastonbury Festival datasets. This is an opinion tweet and doesn't corresponding to any realistic sub-event. The other 4 windows contain noise that is irrelevant to the Glastonbury Festival (talking about Wimbledon Championships, Premier League, etc). Although the amount of noise brought in by the adaptive crawling is less than 1% of the event-relevant traffic [45], the burst detection algorithm still identifies these as abnormal moments (outliers). Although in our previous work we showed that the KwAA by content similarity can quickly recover from non-related event keywords, this still significantly affects the event detection results for these events. Burst detection algorithms can be very sensitive to the sudden spikes caused by the short-lived event non-related keywords. Due to that, the AD dataset gets the lowest event recall. Although the EX suffers from the drawback of irrelevant tweets, as shown in Fig 3, the system framework can identify more realistic sub-event with the help of EX datasets for both events. Consequently, by applying the proposed STRIM framework over all three streams (ALL), both the precision and recall are improved. Although the number of peak windows that corresponding to realistic sub-events is higher, the precision is marginally improved due to the increasing of total number of peak windows. Compared with the detection recall of the BL dataset, the metric for the ALL in the proposed framework is doubled due to the extra amount of event content introduced by the adaptive crawler. As a result, the F1 score is improved as well for the Glastonbury Festival datasets.

**Table 4:** Comparison of event detection between the baseline approach and the proposed framework.

Data stream	Glastonbury Festival			Sochi Olympic		
	P_event_	R_event_	F1	P_event_	R_event_	F1
BL	54.55	45.45	49.59	71.43	55.56	62.50
AD	45.45	36.36	40.40	75	66.67	70.59
EX	64.29	72.73	68.25	83.33	55.56	66.37
ALL	58.62	90.91	71.28	80	100	88.89

The metrics on the Sochi Olympic datasets shows different performance, but all are higher than that of Glastonbury Festival. The reasons for this change are twofold. First, unlike the generic expression about the willingness to go to the festival in the Glastonbury datasets, tweets containing personal feeling in Olympic datasets are always associated with a real-world entity, such as a team or an athlete who is playing the game. Since these tweets are about things reported in newswire, the probability that a peak window is a sub-event is larger. Second, the sub-events in Olympic datasets happen chronologically rather than simultaneously and thus are easier to identify. The zero appearance of the identical sub-event on none of the BL, AD and EX datasets also proves this. Contrary to the Glastonbury Festival, all three metrics on AD dataset are higher than those for the BL dataset. The benefits of additional event content, in this event, can be directly illustrated by comparing the results of AD dataset and BL dataset. While the increase of precision is marginally, the increase in recall is more than 10%, and 18% for the F1 score. This improvement is due to the higher proportion of event-relevant tweets. Namely, no additional noisy windows are detected compared to the results of BL dataset. While these improvements are significant, the proposed parallelized detection framework is still a significant improvement over using either data stream alone. Compared with the results on BL dataset, the precision and recall are improved by 9.57% and 44.44% respectively, and the overall F1 score is improved by 26.39%.

Overall, it can be observed that the adaptive crawling portion of STRIM introduces extra event content that improves the precision and recall on the detection performance. A list of sub-event that is identified by the proposed event monitoring framework for the Glastonbury Festival is illustrated by Table 5. With better understanding of the sub-events of an event of interests, applications require automatic event awareness, such as machine-written journalism and disaster alarming and awareness can be undated.

**Table 5:** List of sub-event by the proposed event monitoring framework (Glastonbury Festival).

Sub-event	Time span	Summary tweet	Descriptive terms
Ben Howard	16:20 to 16:50	@benhowardmusic is some guy #amazing #lovehit	[#amazing] [#jealous] [#wow] [#lovehim] [howard]
Laura Mvula	15:55 to 16:20	Laura Mvula looks stunning! #glastonbury	[heatwave] [#sebheupdate] [laura] [mvula] [6pm]
	18:55 to 19:35	#Glastoshout please stop laura mvula	[#festival] [#jealous] [laura] [manch] [#glastoshout]
Tibetan Monk Throat Singing	16:20 to 17:20	Tibetan monk throat singing……I think you''d have to have been there #glastonbury	[heatwave] [alongside] [#silverhayes] [haircut] [monk]
Elvis Costello	17:20 to 18:05	Olivers Army are on their way #elviscostello #glastonbury	[deborah] [8ish] [hoop] [tenda] [vanessa]
Noah and the Whale	16:20 to 17:20	Noah and The Whale!<3 #glastonbury #wishiwasthere	[noah] [whale] [heatwave] [heading] [#jealous]
	18:55 to 19:55	Noah and the whale #glastonbury #lovethem	[noah] [whale] [belongings] [door] [johnny]
Primal Scream	19:35 to 19:55	The crowd during @screamofficial #stonesglasto #primalscream #therollingstones	[whale] [#primalscream] [noah] [#goodtimes] [#noahandthewhale]
Maverick Sabre	20:25 to 20:55	Maverick Sabre #wow	[maverick] [sabre] [#wow] [wonderwall] [#amazing]
Glastonbury founder supports badger cull	19:55 to 21:15	#glastonbury badger badger badger badger badger badger	[badger] [sabre] [maverick] [wonderwall] [1965]
	21:15 to 21:40	Wonder will Eavis get "BADGERED" tomorrow at #Glastonbury?—"BADGER BADGER BADGER!”	[badger] [switch] [rudiment] [1965] [petition
Two Door Cinema Club	21:10 to 21:35	Two Door Cinema rock and they look like they could do your accounts…#bbcglasto	[cinema] [door] [margaret] [#leftfield] [invite]
Example	21:35 to 22:05	is there anybody, completely off their nut? #example	[#example] [#proud] [#nffc] [#jealous] [cinema]
Rollingstones	22:15 to 23:30	Oh dear the #Stones at #glastonbury look like a Wonga TV ad	[wonga] [#stones] [#glastonbury2013live] [careworker] [#physiotherapy]

## Conclusions

In this paper, we investigate a different perspective that is left unexplored in the current existing literature: the effects and influence of improving the coverage of event content on the sub-event detection task. By applying the statistical-based event detection algorithm to the Twitter stream identified by the adaptive crawler, this paper investigates and proposes a real-time sub-event detection framework before evaluating the framework over two different planned events. Although the characteristics of these two events are different (the Glastonbury Festival contains multiple simultaneous sub-events while the sub-events of the Sochi Olympic are of a more sequential nature), the proposed framework provides better event monitoring, showing significant improvements in both precision and recall.

To conclude, this paper demonstrates that enabling better sub-event detection is achievable by incorporating the burst detection algorithm directly with the adaptive crawling. On the other hand, the experiment results show that while doing a better job than single stream burst detection algorithms, the current sub-event detection algorithm is still insufficient for dealing with events that have multiple sub-events happening simultaneously. In fact, even within the narrow parameters of an event such as a music festival, sports event or disaster scenario, it is very common that sub-events take place simultaneously, however the existing sub-event detection algorithms are tailored for events with sequential sub-events. Our future work will improve our event detection algorithm to enable monitoring of sub-event separately, or even in a hierarchy mode. This would allow us to achieve a better event monitoring by detecting sub-events that overlap in time or occur simultaneously. Finally, we plan to explore the incorporation of different types of event detection algorithms into our framework. The results presented here show sub-event detection in planned scenarios. In unplanned scenarios like disasters and riots, sub-event detection would be very useful for emergency or aid services. However, a limitation of the work we have presented here is the reliance on a burst detection algorithm. Unplanned events typically have large initial bursts which obscure smaller bursts from the detection process.
